# The synchronization of neuronal oscillators determined by the directed network structure of the suprachiasmatic nucleus under different photoperiods

**DOI:** 10.1038/srep28878

**Published:** 2016-06-30

**Authors:** Changgui Gu, Ming Tang, Huijie Yang

**Affiliations:** 1 Business School, University of Shanghai for Science and Technology, Shanghai 200093, China; 2 Web Sciences Center, University of Electronic Science and Technology of China, Chengdu 610054, China; 3 Big data research center, University of Electronic Science and Technology of China, Chengdu 610054, China

## Abstract

The main function of the principal clock located in the suprachiasmatic nucleus (SCN) of mammals is synchronizing the body rhythms to the 24 h light-dark cycle. Additionally, the SCN is able to adapt to the photoperiod of the cycle which varies among seasons. Under the long photoperiod (LP), the synchronization degree of the SCN neurons is lower than that under the photoperiod (SP). In the present study, a potential explanation is given for this phenomenon. We propose that the asymmetrical coupling between the light-signal-sensitive part (the ventralateral part, abbreviation: VL) and the light-signal-insensitive part (the dorsalmedial part, abbreviation: DM) of the SCN plays a role in the synchronization degree, which is reflected by the ratio of the number of the directed links from the VL neurons to the DM neurons to the total links of both directions between the VL and the DM. The ratio is assumed to characterize the directed network structure under different photoperiods, which is larger under the SP and smaller under the LP. We found that with the larger ratio in the situation of the SP, the synchronization degree is higher. Our finding may shed new light on the asymmetrical coupling between the VL and the DM, and the network structure of the SCN.

The principal clock situated in the suprachiasmatic nucleus (SCN) which is composed of ten thousands of neurons in mammals, regulates the circadian rhythms of physiological and behavioral activity^1^,^2^,^3^,^4^. One main function of the SCN is synchronizing the body rhythm to the external 24 h light-dark cycle. The photoperiod of the external 24 h light-dark cycle are altered between seasons especially in the high latitudes, i.e long photoperiod (LP) in the summer and short photoperiod (SP) in the winter. Accordingly, the SCN has evolved to adapt the different photoperiods. Distinct behaviors of the SCN are observed between the LP (e.g. 16 h light: 8 h darkness) and the SP (e.g. 8 h light: 16 h darkness). The neuronal phases are more dispersed under the LP in that the synchronization degree between neurons is smaller compared to the situation of the SP in mice^5^,^6^. This smaller synchronization degree results in the lower entrainment ability of the SCN under the LP^7^. However the mechanism of the altered synchronization degree between different photoperiods has not been clearly discovered so far.

The SCN is a heterogeneous network^8^. A part of the SCN neurons are capable of self-oscillating with intrinsic periods ranging from 22 h to 28 h^9^,^10^,^11^, which are coupled through neurotransmitters and then constitute a network to output a uniform periodic rhythm^12^. The SCN neurons can be classified into two groups structurally and functionally, named the ventralateral part (VL) and the dorsalmedial part (DM) which runs faster than the VL^13^. The VL composed of approximately 25% SCN neurons receives the light information and relays the information to the DM formed by the remaining about 75% SCN neurons in rat^14^,^15^,^16^,^17^. The neurotransmitters which play a role in the coupling are distinct in different regions. The DM neurons produce vasoactive intestinal polypeptide (VIP), the DM neurons secrete arginine vasopressin (AVP)^16^,^17^, and most SCN neurons are sensitive to GABA^18^.

The coupling between the VL and the DM is found to be asymmetrical. Structurally, the asymmetry is due to the dense projections from the VL to the DM and the sparse projections from the DM to the VL, in that the VL dominates the DM^4^. Functionally, several experiments suggest the presence of this asymmetry. In slice, after the removal of the VL, the period of the SCN became short, while the period of the SCN was not altered after the removal of the DM^13^. Another explanation for the asymmetry is that after a jet lag, the VL adapts to the phase (time) of the destination promptly, while the DM requires several days to readjust gradually^15^.

A lot of theoretical works have been motivated to study the network behaviors such as the synchronization degree between the SCN neurons, the rhythmicity of the SCN, the entrainment ability of the SCN and so on^19^,^20^,^21^,^22^,^23^,^24^,^25^,^26^,^27^,^28^,^29^. Most of these works regarded the SCN network as an all-to-all network where each two neuronal nodes are connected. Recently, several works investigated the influence of a small-world network structure of the SCN on the network behaviors^30^,^31^,^32^,^33^. The small-world network structure such as the Newman-Watts(nw) network^30^,^34^ and the Barabási (BA) network^31^,^33^,^35^ improves the synchrony between neurons and strengthens the circadian rhythm of the SCN. The network structure was also taken into account to explain the distinct synchronization degree under different light conditions^32^,^36^,^37^. Refs 32 and 36 suggests that the reduced total number of undirected links between the VL and the DM lead to the smaller synchronization degree under the LP. Nevertheless, no theoretical work has studied the influence of the directed and asymmetrical coupling between the VL and the DM on the network behaviors.

In the present study, an alternative explanation is given for the distinct synchronization degree under different photoperiods by considering the fact of the asymmetrical coupling between the VL and the DM^4^, which is assumed to be reflected by more directed links from the VL to the DM than vice versa in the present study. In order to measure the asymmetrical degree, we define a key parameter as the ratio of the number of directed links from the VL to the DM to the total number of directed links between the VL and the DM. We assumed that the ratio be associated with the duration of photoperiod, i.e. the ratio is larger (smaller) under short (long) photoperiod.

## Description of the Kuramoto Model

Two kinds of models are used to describe the SCN neuronal oscillators, i.e. biochemical models such as Goodwin model^19^,^21^,^22^ and Leloup-Goldbeter model^38^, and phenomenological models such as Kuramoto model^21^,^39^,^40^ and Poincaré model^41^,^42^. One of the simplest biochemical models is the Goodwin model. The Goodwin model takes both the neuronal phase and amplitude into account^19^,^21^,^22^, which is based on the transcription-translation feedback loop in one single neuronal oscillator. The simplest phenomenological mode is the Kuramoto model, which is a generic model and focuses on the phase but not the amplitude^21^,^39^,^40^. Because the amplitude of single neuron does not differ noticeably between the conditions of the LP and SP^5^, we here use the Kuramoto model. The Kuramoto model composed of *N* coupled neurons is described as:


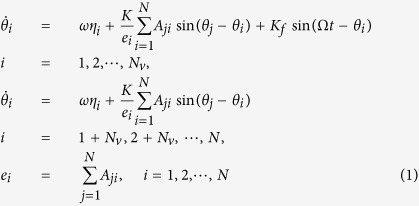


where *θ*_*i*_, *ωη*_*i*_, *K*, *K*_*f*_ and Ω represent the neuronal phase, the neuronal intrinsic frequency, the coupling strength, the light intensity and the frequency of the external light-dark cycle. *N* is the total number of the SCN neuronal oscillators, and *N*_*v*_ and *N* − *N*_*v*_ are the number of neuronal oscillators in the VL and in the DM respectively. We added the light term *K*_*f*_ sin(Ω*t* − *θ*_*i*_) to the VL neuronal oscillators. The parameters are set as follows: *K* = 0.05, 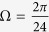, *N* = 500 and *N*_*v*_ = 125. In order to model the intrinsic periods of the uncoupled neuronal oscillator ranging from 22 h to 28 h, the term *ωη*_*i*_ is introduced where *η*_*i*_ satisfies a normal distribution with the mean 1 and the deviation 0.05. Under constant darkness, the period of the SCN network *τ* (the free running period) varies among species which is around but not exactly equal to 24 *h*, for example for human 24.5 h, for chipmunk 24.9 h, for deer mouse 22.9 h and for field vole 23.5 h^3^. For instance we chose 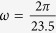 and 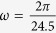. We here assumed that the duration of photoperiod is related to the value of sin(Ω*t*). If the value is larger (smaller) than a predefined value *L*, the time *t* corresponds to light (dark) time. For the 24 h cycle of 12 h light: 12 h dark, the predefined value is 
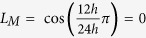
, for the 24 h cycle of 16 h light: 8 h dark (or 8 h light: 16 h dark), the predefined value is 
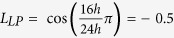
 (or 
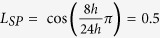
. Thus far, it is unclear whether the influence from the light signal is larger than the influence from other neurons to one neuron or not. Consequently, the value of the light intensity *K*_*f*_ is selected as *K*_*f*_ = 0.5 *K*, *K* and 2 *K*.

**Figure 1:**
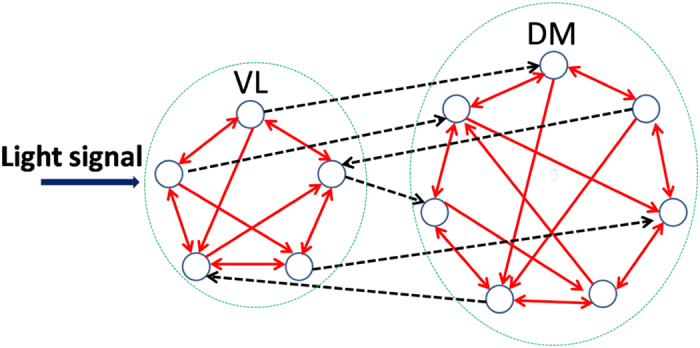
The scheme of the SCN network. The solid arrows represent the directed links within the VL/DM, and the dashed arrows represent the directed links between the VL and DM. Note that, the number of the directed links from the VL to the DM is larger than vice versa.

The network topology is described by a directed adjacent matrix *A*_*ji*_. If there is a directed link from neuronal node *j* to *i*, *A*_*ji*_ = 1; otherwise *A*_*ji*_ = 0. Thus far, the SCN network is suggested to be a small network, yet the details of the SCN network have not been explored. Here, the SCN network is assumed to be a directed Newman-Watts network which is established as follows^34^: at the first step, if the physical distance between two nodes *i* and *j* is smaller than a predefined value *d*, there are directed links *A*_*i*,*j*_ = *A*_*j*,*i*_ = 1 within the VL (DM); at the second step, *j* is directly linked to *i* (*A*_*j*,*i*_ = 1) with possibility *p* within the VL (*i*, *j* = 1, 2, ..., *N*_*v*_) or DM (*i*, *j* = *N*_*v*_ + 1, *N*_*v*_ + 2, ..., *N*); at the third step, the links are from two directions, i.e. *i* (*i* = 1, 2, ..., *N*_*v*_) in the VL is directly linked to *j* (*j* = *N*_*v*_ + 1, *N*_*v*_ + 2, ..., *N*) in the DM ((*A*_*i*,*j*_ = 1)) with possibility *q*_1_, and *j* (*j* = *N*_*v*_ + 1, *N*_*v*_ + 2, ..., *N*) in the DM is directly linked to *i* (*i* = 1, 2, ..., *N*_*v*_) in the VL (*A*_*j*,*i*_ = 1) with possibility *q*_2_. Note that *A*_*i*,*j*_ is not necessarily equal to *A*_*j*,*i*_ in the latter two steps. The parameters are set as: *d* = 2, *p* = 0.05 and *q*_1_ + *q*_2_ = 0.05. The key parameter *δ* is defined as the ratio 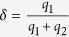, which represents the asymmetrical degree in the number of the directed links from the VL to the DM and that from the DM to the VL. Since the VL dominates the DM and the DM feedbacks to the VL^4^, the range of *δ* is from 0.5 to 1. When the ratio is *δ* = 1, the number of directed links from the DM to the VL is 0; and when the ratio is *δ* = 0.5, the numbers of directed links are equal for both directions. We assume that the case of *δ* close to 1 reflect the network topology under the SP, and the case of *δ* close to 0.5 character the network topology under the LP. A scheme for the SCN network structure is shown in Fig. 1.

In order to explain the distinct synchronization degree of the neuronal oscillators between the LP and the SP observed in experiments^5^, the impact of the asymmetrical degree *δ* on the synchronization degree between the neuron oscillators is studied. The synchronization degree is defined as:


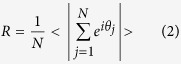


where 〈...〉 represents average over time. *R* is 0 for fully unsynchronized oscillators and 1 for perfect synchronization.

The fourth-order Runge-Kutta method is used for the numerical simulation with time increments of 0.01 h. The initial 1,000,000 time steps were neglected in order to avoid the influence of transients. The initial conditions for each variable were selected randomly from a uniform distribution in the range (0–2*π*) for *θ*. In the supplemental file, the Goodwin model is also taken into account where the results confirm our findings by the Kuramoto model.

## The Effect of the Asymmetrical Degree

**Figure 2:**
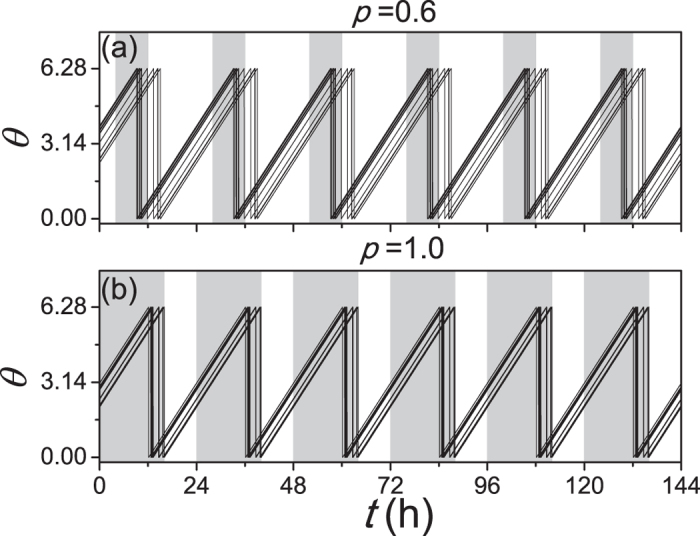
The evolutions of randomly selected neuronal oscillators in the LP (**a**) and the SP (**b**). The asymmetrical degree *p* = 0.6 and 1 correspond to the LP (long photoperiod) and the SP (short photoperiod) respectively. The mean of the neuronal intrinsic periods *τ* is 23.5 h, and the period of the external light-dark cycle is 24 h. The gray (white) area corresponds to the darkness (light).

The illustrative examples for the effect of the asymmetrical degree *δ* on the synchronization degree between neuronal oscillators was shown in Fig. 2. We here used the *δ* = 0.6 (a) and *δ* = 1.0 (b) to mimic the case of the LP and SP respectively. In both the cases of *δ* = 0.6 and 1.0, the randomly selected neuronal oscillators are synchronized to the external cycle, i.e. the phase difference between the oscillators and the cycle is stable. However, the neuronal phases are more dispersed (smaller synchronization degree) with *δ* = 0.6 (LP) than *δ* = 1.0 (SP).

**Figure 3:**
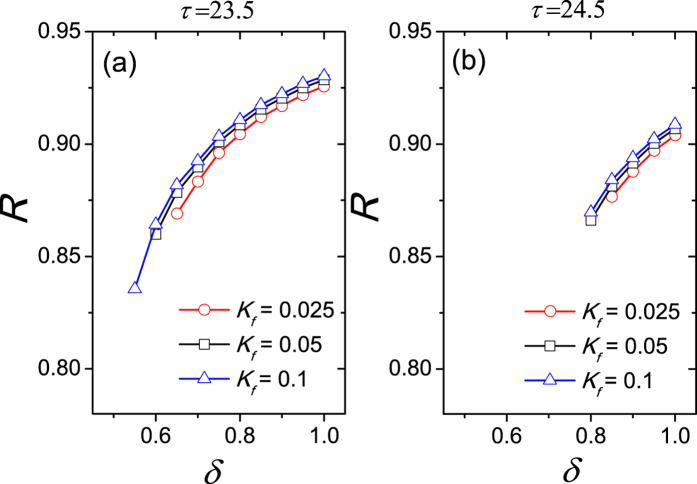
The relationship of the synchronization degree between neuronal oscillators to the asymmetrical degree, when the free running period *τ* is 23.5 h (**a**), and when *τ* is 24.5 h (**b**). *K*_*f*_ represents the light intensity and the coupling strength is *K* = 0.05. The synchronization degree *R* is not calculated when the neurons are not entrained to the external 24 h cycle. The intrinsic periods are not distinct in the VL and the DM.

Further the relationship between the synchronization degree *R* and the asymmetrical degree *δ* was examined in Fig. 3. Two values of free running periods *τ* are considered, when *τ* is smaller than 24 h (a) and *τ* is larger than 24 h (b). Since the comparison between the influence from light and the influence from the other neurons to one neuron is unknown, we tested three cases of the light intensity, i.e. *K*_*f*_ < *K*, *K*_*f*_ = *K* and *K*_*f*_ > *K*. The synchronization degree *R* is calculated when the SCN network is entrained to the external SP or LP cycle, i.e. the periods of the neuronal oscillators are equal to 24 h. In both panels, when *δ* is close to 0.5, the SCN network is not entrained to the external cycle for most light intensity *K*_*f*_. When *δ* is close to 1, the SCN network is entrained to the external cycle for each light intensity *K*_*f*_. With the increase of *δ* the synchronization degree *R* increases for each light intensity *K*_*f*_.

**Figure 4:**
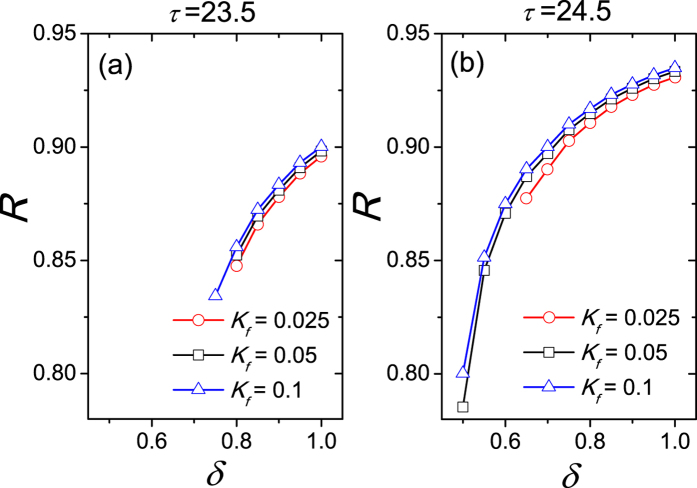
The relationship of the synchronization degree between neuronal oscillators to the asymmetrical degree, when the free running period *τ* is 23.5 h (**a**), and when *τ* is 24.5 h (**b**). The neurons in the DM run faster than that in the VL. This figure corresponds to Fig. 2.

In order to mimic the DM running faster^13^, let the the mean of *η*_*i*_, *i* = 1, 2, ..., *N*_*v*_, multiply a factor 0.985 and the the mean of *η*_*i*_, *i* = 1 + *N*_*v*_, 2 + *N*_*v*_, ..., *N*, multiply a factor 1.005. As a consequent, the mean of *η*_*i*_, *i* = 1, 2, ..., *N*, remains 1 in that the free running period *τ* of the SCN is not altered, but the intrinsic period of the DM is 0.5 h smaller than it of the VL. The relationship between the synchronization degree *R* and asymmetrical degree *δ* was examined in Fig. 4 with the DM neurons running faster than the VL neurons. In Accordance with Fig. 3, we observed that the synchronization degree *R* increases with the increase of *δ* in both cases of *τ* > 24 (a) and *τ* < 24 (b).

**Figure 5:**
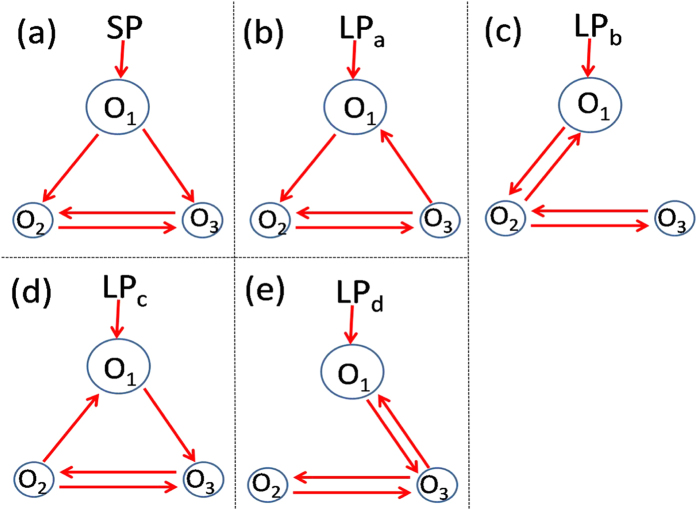
The network topologies for three neuronal nodes. ‘O_1_’ is located in the VL which receives the light signal, and ‘O_2_’ and ‘O_3_’ are situated in the DM, where ‘O’ means oscillator. The SP corresponds to the zero link from the DM to the VL (**a**), and the LP corresponds to the equal number of links from the DM to the VL and from the VL to the DM (**b–e**). The size of the cycle represents the length of neuronal intrinsic period.

Next to the numerical simulations, we performed the analytical analysis. For simplicity, the number of neuronal oscillators is set as *N* = 3, where one is in the VL with larger intrinsic period and the other two are in the DM, and two directed links between the VL and the DM (Fig. 5). When both two directed links are from the VL to DM, the asymmetrical degree is *δ* = 1 which reflects the network topology of the SCN under the SP (a). When there is one link in each direction, the asymmetrical degree is *δ* = 0.5 which characters the network topology of the SCN under the LP (b-e). Without losing generality, let oscillators ‘2’ and ‘3’ be linked to each other, and their periods be the same (*ω*_3_ = *ω*_2_). The Kuramoto model, Eq. 1, composed of three oscillators based on the network structure in Penal (a) can be written as:


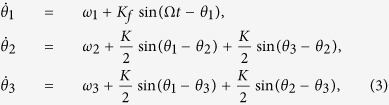


When the network is entrained to the external cycle, the periods of the neurons are equal to the period of the external cycle (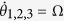). Consequently, the phase difference between each two neuronal oscillators and the phase difference of each oscillator to the external cycle are unchangeable. Thus, we defined *ψ* = Ω*t* − *θ*_1_, *ϕ*_12_ = *θ*_1_ − *θ*_2_, *ϕ*_32_ = *θ*_3_ − *θ*_2_ and *ϕ*_13_ = *θ*_1_ − *θ*_3_ which determine the synchronization degree. From the first equation of Eq. 3, we obtained 
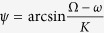
. Because the neurons 2 and 3 are identical, *ϕ*_23_ = *ϕ*_32_ = 0. From the second or third equation of Eq. 3, we obtained 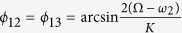.

**Figure 6:**
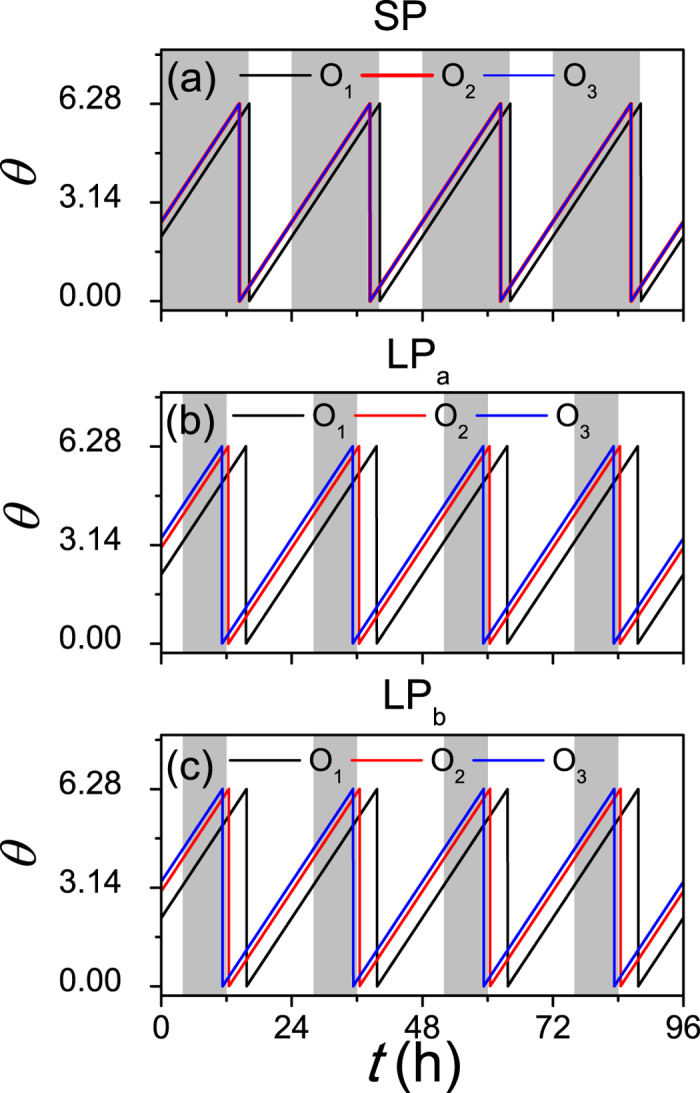
The evolutions of neuronal oscillators in the SP (**a**) and the LP (**b,c**) with *N* = 3. The network structures of (**a–c**) are introduced as in (**a–c**) of Fig. 5 respectively. ‘O_1_’ is located in the VL which receives the light signal, and ‘O_2_’ and ‘O_3_’ are situated in the DM, where ‘O’ means oscillator. The free running period is *τ* = 23.5, the coupling strength is *K* = 0.1 and the light intensity is *K*_*f*_ = 0.1. The gray (white) area corresponds to the darkness (light).

Similarly, the phase difference can be found for the network structures of the SCN under the LP described in Fig. 5(b–e). Because oscillators ‘2’ and ‘3’ are identical, (d) and (e) are symmetrical cases for (b) and (c) respectively. Therefore, (b) and (c) are selected for the analysis. Interestingly, we found that the phase differences are not altered between (b) and (c), which are 
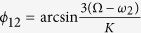
, 
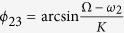
 and 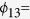 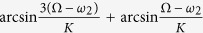. Thus, the absolute values of *ϕ*_12_, *ϕ*_23_ and *ϕ*_13_ are evidently smaller in the SP than in the LP, in that the synchronization degree is smaller in the SP. The theoretical results were confirmed by our numerical simulations (Fig. 6).

## Conclusion and Discussion

In the present study, an alternative explanation was given for the distinct synchronization between the SCN neurons under different photoperiods by considering the asymmetrical coupling between the light-signal-sensitive VL part and the light-signal-insensitive DM part^4^. This asymmetrical coupling is characterized by the assumption of more directed links from the VL to the DM than from the DM to the VL in the present study. In order to determine the asymmetrical degree, we defined the ratio of the number of the directed links from the VL neurons to the DM neurons to the total links between the VL and the DM. The ratio (asymmetrical degree) is assumed to reflect the directed network structure under different photoperiods, which is larger under the SP and smaller under the LP. We found that with the larger ratio in the situation of the SP, the synchronization degree is higher.

Recently, it has found that not all the SCN neurons are capable of self-oscillating. After the neurons are uncoupled from the SCN network, three oscillatory phenotypes for the SCN neurons are observed: sustained oscillations, damped oscillations, and arrhythmic patterns^11^,^32^,^43^,^44^. Nevertheless, the Kuramoto model focus on the phase and is in lack of amplitude information. In order to pursue to model the nonrhythmicity, the Goodwin model is studied in the supplemental file, where independent of the oscillatory phenotypes, the synchronization degree is also found to be determined by the asymmetrical degree which reflects the photoperiod.

Most previous theoretical works regarded the SCN as an all-to-all network for simplicity. Very recently, the network structure of the SCN was found to characterize the small-word properties in experiment^45^, yet the details of the network structure have not been found so far. Several theoretical work investigated the effect of the small-world network structure which was found to improve the synchronization degree and the amplitude of the rhythms^30^,^31^,^32^,^33^,^36^,^37^,^44^. In refs 32 and 36, the reduction of the synchrony between the SCN neurons under the LP was suggested to be due to the decreased number of undirected links between the VL and DM. However, as best as we know, no work has considered the directed network structure of the SCN, where the directed links from the VL to the DM should be evidently more than vice versa. As a first step, the directed network structure was investigated to explain the distinct synchronization between different photoperiods in the present study, but the directed network structure may also help to understand the other network behaviors of the SCN, such as the ‘dissociation’ phenomenon between the VL and the DM under a 22 h light-dark cycle^46^ and the ‘split’ phenomenon between the VL and the DM under constant light^47^. At last, we hope our present study could help understanding the synchronization phenomenon in other neuronal network^48^,^49^,^50^.

## Additional Information

**How to cite this article**: Gu, C. *et al.* The synchronization of neuronal oscillators determined by the directed network structure of the suprachiasmatic nucleus under different photoperiods. *Sci. Rep.* **6**, 28878; doi: 10.1038/srep28878 (2016).

## Supplementary Material

Supplementary Information
