# Supplementary material for: The synchronization of neuronal oscillators determined by the directed network structure of the suprachiasmatic nucleus under different photoperiods
Source: Sci Rep. 2016 Jun 30;6:28878. doi: 10.1038/srep28878 (PMC4928114; doi:10.1038/srep28878)
Supplement: Supplementary Information [file srep28878-s1.pdf]

# The synchronization of neuronal oscillators determined by the directed network structure of the suprachiasmatic nucleus under different photoperiods

Changgui Gu[1*], Ming Tang[2;3], Huijie Yang[1].

[1]Business School, University of Shanghai for Science and Technology, Shanghai 200093, China

[2]Web Sciences Center, University of Electronic Science and Technology of China, Chengdu 610054, China

[3]Big data research center, University of Electronic Science and Technology of China, Chengdu 610054, China

*Corresponding author: gu_changgui@163.com

## Supplementary information

### Description of the Goodwin model

The Goodwin model is widely used to describe the circadian oscillators[1,2]. The Goodwin model composed of $N$ neuronal oscillators is shown in below:

$$\begin{aligned}
\frac{dx_i}{dt} &= \sigma_i(\alpha_1 \frac{k_1^n}{k_1^n + z_i^n} - \alpha_2 \frac{x_i}{k_2 + x_i}) + \alpha_c \frac{gf_i}{k_c + gf_i} + L_i \\
\frac{dy_i}{dt} &= \sigma_i\left(k_3 x_i - \alpha_4 \frac{y_i}{k_4 + y_i}\right) \\
\frac{dz_i}{dt} &= \sigma_i\left(k_5 y_i - \alpha_6 \frac{z_i}{k_6 + z_i}\right) \\
\frac{dV_i}{dt} &= \sigma_i\left(k_7 x_i - \alpha_8 \frac{V_i}{k_8 + V_i}\right) \\
f_i &= \frac{1}{e_i}\sum_{j=1}^{N} A_{ji} V_j, \quad e_i = \sum_{j=1}^{N} A_{ji} \\
i &= 1,2,....,N
\end{aligned} \tag{S1}$$

where the variables $x$, $y$ and $z$ which represent the gene mRNA, protein and the inhibitor respectively, constitute a negative feedback loop in one neuronal oscillator. The individual oscillators are coupled through the local mean field $f$ of the transmitter $V$ produced by the gene mRNA. The local mean field $f$ of $i_{th}$ oscillators is defined as the mean for the transmitter from the neighbors of the oscillator $i$. The matrix $A_{ij}$ describes the topology of the SCN network as it in the Kuramoto model, and $e_i$ represents the number of the neighbors of oscillator $i$. We assumed that the network topology is altered between different photoperiod, i.e. the number of the directed links from the VL to the DM and from the DM to the VL is altered (the details

please see the main text). The light term $L_i$ is defined as:

$$\begin{cases} if \ \ \mathrm{mod}(t,24) > T_L \text{ and } i \le N/4, & L_i = K_f \\ else, \ L_i = 0 \end{cases} \quad \text{(S2)}$$

Where the parameter $T_L$ is the photoperiod, and $K_f$ is the light intensity. The term $i \le N/4$ corresponds to the fact that about *25%* SCN neurons are sensitive to light signal.

In order to model the diversity of the intrinsic periods of the neurons, the factor $\sigma_i$ is introduced which satisfies a normal distribution with mean 1 and the deviation 0.05. The coupling strength *g* is 0.5, the light intensity $K_f$ is 0.01, the number of neurons is *N* =400 and the other parameters are the same as in Ref.[1],

$$\begin{aligned} &\alpha_1 = 0.7nM/h,\ k_1 = 1nM,\ n = 4,\ \alpha_2 = 0.35nM/h,\ k_2 = 1nM, k_3 = 0.7/h, \\ &\alpha_4 = 0.35nM/h,\ k_4 = 1nM,\ k_5 = 0.7/h,\ \alpha_6 = 0.35nM/h, k_6 = 1nM,\ k_7 = 0.35/h, \\ &\alpha_8 = 1nM/h,\ k_8 = 1nM, \alpha_c = 0.4nM/h,\ k_c = 1nM \end{aligned}$$

To mimic the nonrhythmic compartments in the VL as observed in experiments[3], the parameter *n* and $\alpha_2$ is set as 3 and 0.5 respectively for all the VL neurons as in Ref.[4]. The mark for one neuronal oscillator, the VL, the DM and the DM are selected as $V_i$, $F_{VL}$, $F_{DM}$ and *F* respectively, where

$$F_{VL} = \frac{1}{N/4}\sum_{i=1}^{N/4} V_i, \qquad F_{DM} = \frac{1}{3N/4}\sum_{i=N/4+1}^{N} V_i, \qquad F = \frac{1}{N}\sum_{i=1}^{N} V_i \qquad \text{(S3)}$$

The synchronization degree between neuronal oscillators is defined as[4]:

$$R = \frac{\langle F^2 \rangle - \langle F \rangle^2}{\frac{1}{N}\sum_{i=1}^{N}\left(\langle V_i^2 \rangle - \langle V_i \rangle^2\right)} \qquad \text{(S4)}$$

where < > denotes the average over time. If the synchronization degree is *R* =1/*R* =0, the neuronal oscillators are perfectly synchronized/unsynchronized. The network amplitude is $\rho = F_{peak} - F_{trough}$. In the follows, we will examine the alternation of the synchronization degree *R* and the network amplitude $\rho$ with the distinct photoperiods.

## Simulation results based on the Goodwin model

Figure S1 shows the evolutions of 10 randomly selected neuronal oscillators under short and long photoperiods when the VL neurons are nonrhythmic. Under the LP (long photoperiod), the neuronal phases are more dispersed, and the neuronal amplitudes are also smaller than those under the SP (long photoperiod) (a and b). Due to the dispersed neuronal phases and the smaller neuronal amplitude, the amplitude of the SCN network is smaller under the LP (c).

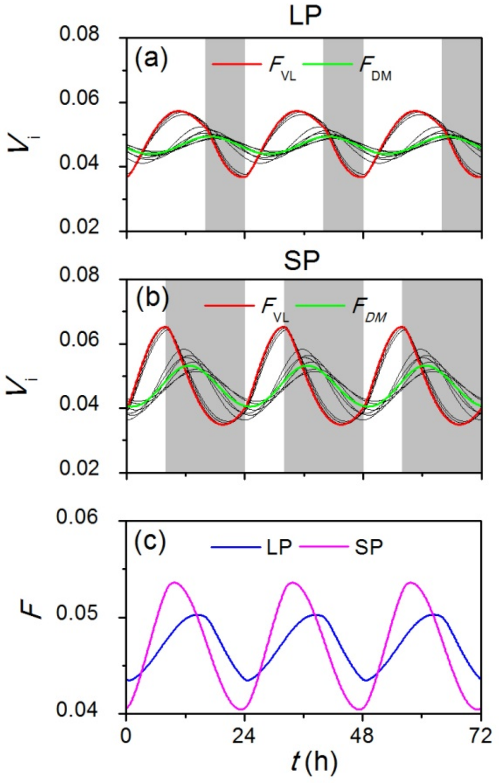


Fig S1. Te evolutions of 10 randomly selected neuronal oscillators under different photoperiods when the VL neurons are nonrhythmic. Under short period (a) and long photoperiod (b). $F_{VL}$, $F_{DM}$ and $F$ represent the mean of the transmitters $V_i$ of the VL neurons, the DM neurons and the SCN neurons respectively. LP and SP represent the long photoperiod (16 h light: 8 h dark ) and the short photoperiod (8 h light: 16 h dark ) respectively. The grey region corresponds to darkness.

Next the relationships of the degree $R$ and the network amplitude $\rho$ to the photoperiod are examined in Fig. S2. Consistent with the observation in Kuramoto model, the synchronization degree $R$ (a) decreases with increase of the photoperiod when the VL neurons are either rhythmic or nonrhythmic. In addition, the network amplitude $\rho$ also decreases with increase of the photoperiod whereas the

Kuramoto model is in lack of amplitude information.

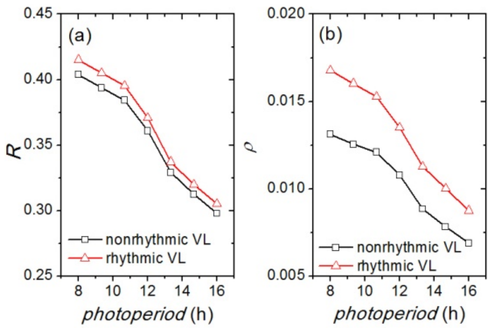


Fig S2. The relationships of the degree $R$ (a) and the network amplitude $\rho$ (b) to the photoperiod. Both the situations of the VL neurons being nonrhythmic and rhythmic are examined.

Here, the observation in the Goodwin model confirms our main result from the Kuramoto model, i.e. the synchronization degree is larger under SP than it under LP, since the number of directed links from the VL to the DM are more under SP than it under LP. In addition, we observed that the amplitude of the SCN network is improved under SP, while in the Kuramoto model the amplitude information cannot be obtained. Our results of the Goodwin model are in accordance with the findings in experiments where both the larger amplitude of the SCN network and the higher synchronization degree under SP are observed[5].

## References:


1 Gonze, D., Bernard, S., Waltermann, C., Kramer, A. & Herzel, H. Spontaneous synchronization of coupled circadian oscillators. *Biophys. J.* **89**, 120-129 (2005).

2 Gu, C., Wang, J. & Liu, Z. Free-running period of neurons in the suprachiasmatic nucleus: Its dependence on the distribution of neuronal coupling strengths. *Phys. Rev. E* **80**, 030904 (2009).

3 Westermark, P. O., Welsh, D. K., Okamura, H. & Herzel, H. Quantification of circadian rhythms in single cells. *PLoS Comput. Biol.* **5**, e1000580 (2009).

4 Bodenstein, C., Gosak, M., Schuster, S., Marhl, M. & Perc, M. Modeling the seasonal adaptation of circadian clocks by changes in the network structure of the suprachiasmatic nucleus. *PLoS. Comput. Biol.* **8**, e1002697 (2012).

5 VanderLeest, H. T. *et al.* Seasonal encoding by the circadian pacemaker of the SCN. *Curr. Biol.* **17**, 468-473 (2007).
